# Effect of Noni (*Morinda citrifolia* Linn.) Fruit and Its Bioactive Principles Scopoletin and Rutin on Rat Vas Deferens Contractility: An *Ex Vivo* Study

**DOI:** 10.1155/2014/909586

**Published:** 2014-06-22

**Authors:** Vijayapandi Pandy, Megala Narasingam, Thubasni Kunasegaran, Dharmani Devi Murugan, Zahurin Mohamed

**Affiliations:** Department of Pharmacology, Faculty of Medicine, University of Malaya, 50603 Kuala Lumpur, Malaysia

## Abstract

This study examined the effect of methanolic extract of *Morinda citrifolia* Linn. (MMC) and its bioactive principles, scopoletin and rutin, on dopamine- and noradrenaline-evoked contractility in isolated rat vas deferens preparations. MMC (1–40 mg/mL), scopoletin (1–200 *μ*g/mL), and rutin hydrate (0.6–312.6 *μ*g/mL) dose-dependently inhibited the contractility evoked by submaximal concentrations of both dopamine and noradrenaline, respectively. Haloperidol and prazosin, reference dopamine D_2_, and *α*_1_-adrenoceptors antagonists significantly reversed the dopamine- and noradrenaline-induced contractions, respectively, in a dose-dependent manner. Interestingly, MMC *per se* at higher doses (60–100 mg/mL) showed dose-dependent contractile response in rat vas deferens which was partially inhibited by high doses of haloperidol but not by prazosin. These results demonstrated the biphasic effects of MMC on dopaminergic system; that is, antidopaminergic effect at lower concentrations (<40 mg/mL) and dopaminergic agonistic effect at higher concentrations (>60 mg/mL). However, similar contractile response at high doses of scopoletin (0.5–5 mg/mL) and rutin hydrate (0.5–5 mg/mL) *per se* was not observed. Therefore, it can be concluded that the bioactive principles of MMC, scopoletin, and rutin might be responsible for the antidopaminergic and antiadrenergic activities of MMC.

## 1. Introduction

*Morinda citrifolia* Linn. (family: Rubiaceae) is also known as noni, Indian mulberry, hog apple, and cheese fruit. The fruit, flower, leaves, bark, and root of* M. citrifolia* have been used for diverse medicinal purposes as traditional plant-based medicine. In fact, the fruits of this plant have benefited the Polynesians over 2000 years as an alternative medicine for antibacterial, antiviral, antifungal, antitumor, anthelmintic, analgesic, hypotensive, anti-inflammatory, and immune-enhancing effects [1].

The decoction or infusions of roasted mature unripe fruits have been recommended to relieve the symptoms of nausea and vomiting [2]. It has also been reported that noni fruit extract showed prokinetic and antiemetic activity on the intestinal transit being delayed by apomorphine (a potent agonist of dopamine D_2_ receptor) in mice and on the apomorphine-induced emesis in dogs, respectively. These actions are attributed to antidopaminergic effect of noni fruit extract [3]. In contrast, Pu et al. (2004) found that gastric emptying was significantly inhibited in rats which were treated with noni juice (0.25, 1, or 4 mL/kg) for 7 days [4]. These opposing effects on gastric emptying observed for noni by two different research groups might be due to the difference in the type of fruit extract and the concentration used in these experiments [5].

Our recent report revealed that the acute treatment of methanolic extract of* M. citrifolia* (MMC 1, 3, 5, and 10 g/kg, p.o.) significantly decreased the apomorphine-induced cage climbing behaviour and methamphetamine-induced stereotypy behaviour in mice in a dose-dependent manner which demonstrated the antidopaminergic effect of noni fruit extract [6]. Furthermore, it was also reported that the administration of* M. citrifolia* fruit extract significantly increased the brain levels of serotonin, dopamine, and noradrenaline in rats [7]. Similarly, other neurochemical analysis of noni-treated rats (1 mL/day, p.o.) for 15 days revealed significant changes in noradrenergic, dopaminergic, or serotonergic systems in amygdala, hippocampus, and substantia nigra [8]. Besides that, in some preliminary studies noni's capability in lowering blood pressure [9] and its vasodilatory properties [10] have been reported. However, the possible modes of action(s) of noni on the cardiovascular system are lacking [11].

Studies have shown that noni fruit contains a number of active constituents including rutin (3,3′,4′,5,7-pentahydroxyflavone-3-rhamnoglucoside), a flavonoid and scopoletin (6-methoxy-7-hydroxycoumarin), a coumarin derivative. Rutin has been reported to exert numerous pharmacological activities including antioxidant, anticarcinogenic, neuroprotective, and anticonvulsant activity [12–15]. Scopoletin has been reported to exhibit antioxidant, analgesic, and anti-inflammatory effects [16, 17]. In addition, scopoletin has been recommended as a marker constituent for the quality control and pharmacokinetic study of noni products [18]. It has also been demonstrated that scopoletin and rutin, respectively, showed antidepressant activity mediated by its interaction with *α*_1_- and *α*_2_-adrenoceptors [19, 20]. However, there has hitherto been no report demonstrating the interaction of rutin and scopoletin on the dopaminergic system.

Thus, the present study aimed to investigate the intervention of noni (*Morinda citrifolia* Linn.) and its bioactive principles, scopoletin and rutin, on the dopaminergic and noradrenergic systems using isolated rat vas deferens preparations.

## 2. Materials and Methods

### 2.1. Plant Collection and Identification

The fresh unripe fruits (dark green) of* M. citrifolia* were collected in January 2012 from Malacca, Malaysia. The plant material was taxonomically identified and authenticated by Rimba Ilmu, Institute of Biological Sciences, University of Malaya, and the voucher specimen (KLU 47738) was deposited at Rimba Ilmu for future reference. The fruits were cut into thin slices and shade dried. The shade dried fruit slices were pulverized in a mechanical grinder to obtain a coarse powder.

### 2.2. Preparation of* M. citrifolia* Fruit Extract

The methanolic extract of* M. citrifolia* (MMC) was prepared using cold extraction with sonication. The coarse fruit powder (1.8 kg) was soaked with 10L of methanol (Scharlau, Spain, isocratic HPLC grade) for 20 h followed by sonication using a water bath sonicator at 30°C for another 4 h. The resultant solution was evaporated under vacuum in a rotary evaporator to obtain a dry mass extract (yield: 14.36% w/w). The dried solvent-free crude methanolic extract of* M. citrifolia* was stored at 4°C in an air-tight, labelled, amber-coloured container until further use.

### 2.3. Chromatography Conditions and Instrumentation

MMC prepared as above was subjected to 1100 HPLC instrument (Agilent Technologies, California, USA) equipped with auto sampler and a fraction collector. Chromatographic separations were carried out on an Agilent Zorbax ODS C18 (4.6 × 250 mm, 5 *μ*m, 70 A) and column temperature was maintained at 25°C. Mobile phase composed of methanol and 1.0% acetic acid in water was used. Elution was performed with a flow rate of 1 mL/min. The injection volume was 30 *μ*L and the detection wavelength was 365 nm. Prior to injection, all samples were filtered through 0.20 *μ*m syringe filter into HPLC sample vial to remove any remaining particles.

HPLC-purified MMC was further subjected to LCMS analysis. The system comprised of Agilent 1290 Infinity LC system coupled to Agilent 6520 Accurate-Mass Q-TOF mass spectrometer with dual ESI source used to analyse the samples. The LC separation was carried out by an Agilent Zorbax SB-C18 Narrow-Bore (2.1 × 150 mm, 3.5 *μ*m). LC parameters are autosampler temperature, 25°C; injection volume, 0.5 *μ*L; column temperature, 25°C; and flow rate of 0.21 mL/min. A gradient system composed of solvents A (0.1% formic acid in water) to B (0.1% formic acid in 100% methanol) was employed. Mass spectra data were acquired using the following settings: ESI capillary voltage was set at 4000 V (+) ion mode and 4000 V (−) ion mode and fragmentor at 125 V. The liquid nebulizer was set to 45 psig and the nitrogen drying gas was set to a flow rate of 10 L/min. Drying gas temperature was maintained at 300°C. The vaporizer temperature was maintained at 300°C. The ionization interface was operated in both positive and negative modes. Data was acquired at a rate of 1.03 spectra/s with a stored mass range of 100–3200* m/z* for positive mode and 115–3200* m/z* for negative mode. Data was collected using Agilent MassHunter Workstation Data acquisition software. LCMS data files were processed by Agilent MassHunter Qualitative Analysis B.05.00 software. Feature finding was achieved using the molecular feature extraction (MFE) and correlation algorithms, which locates the groups of covariant ions in each chromatogram. In positive-ion mode this included adducts (H^+^, Na^+^, K^+^, and +NH_4_) and in negative ion mode it included adducts (−H and +Cl).

#### 2.3.1. Tentative Compound Identifications

The Agilent METLIN (METLIN_AM_PCDL-N-130328.cbd) database was used to make tentative identifications from the mass lists created in Agilent Mass Profiler software. This database includes masses, chemical formulas, and structure information for various compounds. All features were searched against the METLIN database according to match* m/z* value. A list of identified compounds was generated.

#### 2.3.2. Quantification of Scopoletin and Rutin in MMC by LCMS/MS Analysis

Primary stock solution of rutin and scopoletin standard was prepared by dissolving the compounds in methanol to achieve the desired concentration of 0.5 mg/mL. Working standards solution was prepared by serial dilution of primary stock solution using methanol. Peak area was plotted against the corresponding concentrations to obtain calibration plot. A MMC stock solution was prepared at the concentration of 10 mg/mL in methanol. All the samples prepared were filtered through a 0.22 nylon filter (Bioflow, Malaysia) and were transferred to autosampler vial for LCMS/MS quantification analysis. The concentration of each constituent was expressed in *μ*g/mg of dry samples.

The LCMS/MS quantification analysis was performed using Agilent 1290 infinity UHPLC, coupled with Agilent 6410 Triple quad LCMS. The mass detector was equipped with electrospray ionization (ESI) interface and controlled by MassHunter software. 2 *μ*L of all the samples prepared was loaded on a 2.1 mm (*i.d*) Narrow-BoreSB-C18 (length 150 mm) analytical column (particle size 3.5 mM) used with a flow rate of 0.5 mL/min in a solution A (0.1% formic acid in water) and solution B (100% acetonitrile with 0.1% formic acid). The total gradient time for the LCMS run was 11 min. The ionization conditions were adjusted at 350°C and 4000 V for capillary temperature and voltage, respectively. The nebulizer pressure was 45 psi and the nitrogen flow rate was 11 L/min.

### 2.4. Animals

Sprague-Dawley male rats (250–300 g), obtained from the laboratory animal centre, University of Malaya, were used in this study. The rats were housed in polycarbonate cages in a group of four animals under standard laboratory conditions at temperature of 22 ± 1°C and 12/12 h light/dark cycle (light on from 8 am to 8 pm). Animals were fed with standard laboratory food pellets and water* ad libitum*. Animal Care and Use Committee, University of Malaya, Kuala Lumpur, approved the experimental protocol (ACUC Ethics number FAR/27/01/2012/PV (R)) and care of the animals was taken as per guidelines of the Council for International Organization of Medical Sciences (CIOMS) on animal experimentation [21].

### 2.5. Drugs and Chemicals

Dopamine hydrochloride, L-noradrenaline, prazosin hydrochloride, haloperidol, rutin hydrate, dimethyl sulfoxide (Sigma-Aldrich), and scopoletin (Friendemann Schmidt Chemicals) were used. All the drug solutions were prepared fresh in double distilled water. Dopamine hydrochloride and L-noradrenaline were dissolved in double distilled water containing ascorbic acid (0.125% w/v). The stock solution of different concentrations of MMC, rutin hydrate, and scopoletin was prepared using DMSO (0.1% v/v). All reagents were of isocratic HPLC grade unless stated otherwise. HPLC grade water was prepared by reverse osmosis and further purified.

### 2.6. Effect of MMC, Scopoletin, and Rutin Hydrate on Dopamine or Noradrenaline-Evoked Contractile Response in Isolated Rat Vas Deferens

The rats were sacrificed by CO_2_ inhalation and pairs of vas deferens were dissected out and fixed on a wax plate with fine nail. After carefully removing blood vessels and connective tissues, the epididymal portions of the vas deferens (length, 1 cm) were cut from the preparation, mounted with silk thread on to a hook in a 10 mL organ bath, and immersed in Krebs-Henseleit (K-H) solution gassed with 95% O_2_ plus 5% CO_2_ to achieve a pH of 7.3-7.4; the temperature was 37°C. Preparations were stretched to a preload tension of 500 mg and equilibrated for 60 min during which time the bath solution was changed every 15 min. Isometric tension was recorded by a force transducer (Grass Instrument Co., Quincy, MA, USA) and the output was amplified and recorded continuously using the PowerLab recording system (AD Instruments, Sydney, Australia). The K-H solution contained (mM) NaCl 119, KCl 4.7, CaCl_2_ 2.5, MgSO_4_ 1.0, NaHCO_3_ 25, KH_2_PO_4_ 1.2, EDTA 0.03, and D-glucose 11.1 as previously described [22]. After the equilibration period, the contractile responses of vas deferens tissues were tested for viability by the repeated addition of high KCl solution (high K, 80 mM).Once reproducible contractions were obtained with high K, a full noncumulative dose-response curve was obtained by a stepwise increase in concentrations of the agonist (noradrenaline (NA) or dopamine (DA)). DA (0.4–102.4 *μ*g/mL) and NA (0.1–12.8 *μ*g/mL) were tested. Each dose was applied for 30s and then washed thoroughly (at least three times) before the next higher dose was added [23].

From the dose-response curves of DA/NA, a submaximal dose of each agonist was selected for further studies. In the presence of DMSO (0.001% v/v) or the various concentrations of MMC (1–40 mg/mL, 5 min incubation), scopoletin (1–200 *μ*g/mL, 5 min incubation), and rutin hydrate (0.6–312.6 *μ*g/mL, 5 min incubation) in ascending order, the contractile response of the chosen submaximal dose of DA/NA was recorded. Decrease in response with respect to the vehicle control (DMSO) represents inhibitory effect of the extract/bioactive compounds on the corresponding agonist. The % contraction of the chosen submaximal dose of DA (25.6 *μ*g/mL) or NA (3.2 *μ*g/mL) over high KCl solution (high K, 80 mM) in presence of DMSO/MMC/scopoletin/rutin hydrate was calculated. The same set of experiments was also repeated in presence of reference drugs, haloperidol (dopamine D_2_-receptor blocker) and prazosin HCl (*α*_1_-adrenoceptor blocker) as positive controls.

In order to examine the nature of antagonism (reversible or irreversible) by MMC on DA/NA-evoked contractile response in isolated rat vas deferens, log dose-response curves of DA/NA in absence and presence of MMC (1–20 mg/mL, 10 min incubation) were carried out. Similarly, the effect of MMC (1–40 mg/mL, 10 min incubation) on high K- (80 mM) evoked contractility in rat vas deferens was also studied.

In a separate experiment, contractile response of MMC (20–100 mg/mL)* per se* was tested. The contractile response of MMC* per se* was recorded for 5 min and washed thoroughly with fresh K-H solution (at least three times in 5 min interval) before the next higher dose of MMC was added. Following that, contractile response of submaximal dose of MMC (80 mg/mL) was recorded in presence of prazosin HCl (0.01, 0.1, and 1.6 *μ*g/mL) and haloperidol (12.8, 128, and 500 *μ*g/mL), respectively. Similarly, the contractile responses of scopoletin (0.5–5 mg/mL) and rutin hydrate (0.5–5 mg/mL)* per se* on rat vas deferens were also studied.

### 2.7. Statistical Analysis

The concentrations indicated in the text or in the figures represent the final tissue-bath concentration of the respective drugs. The responses were recorded as mean ± standard error of mean (S.E.M) and “*n*” indicates number of rats used for each set of data. The statistical significance of differences between treatments was evaluated by unpaired Student's *t*-test when comparing means of two groups and one-way analysis of variance (ANOVA) and Dunnett post hoc test for more than two group comparisons. A value of *P* < 0.05 was considered as statistically significant.

## 3. Results

### 3.1. Phytochemical Characterization of the MMC

**Figure 1:**
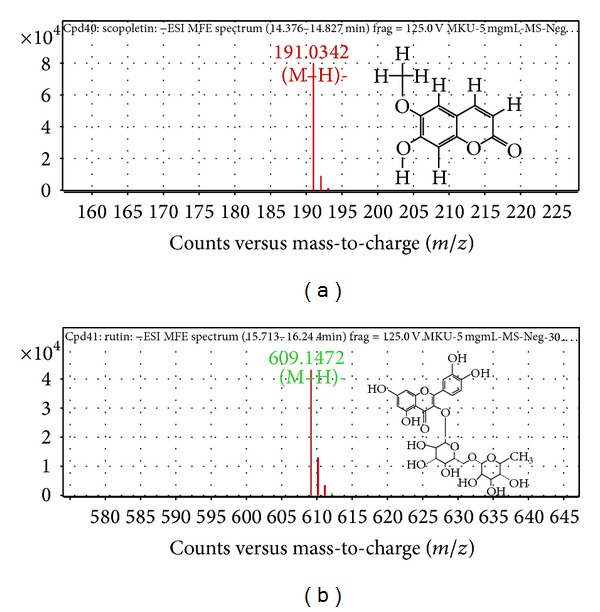
MFE zoomed mass spectrum of (a) scopoletin and (b) rutin obtained from MMC.

MS parameters were optimized by individual standards of 10 mg/L of target compounds that were injected by infusion in full scan in positive and negative modes. For scopoletin, ESI negative ion mode provided the best response where unprotonated molecular ion [M-H]^−^ peak was observed at* m/z* 191.0342 (Figure 1(a)). Rutin was detected only in negative mode. The unprotonated molecular ion [M-H]^−^ peak of rutin was observed at* m/z* 609.1472 (Figure 1(b)).

The other components identified in full scan in positive modes are* monosaccharides*: D-glucoheptose, fagomine, and L-galactose;* disaccharides*: D-(+)-cellobiose and palatinose;* polysaccharides*: levan and hyaluronic acid;* glycosides*: paromomycin, gentamicin C1a, netilmicin, and antibiotic JI-20A;* sugar acid*: isovalerylglucuronide;* sugar alcohol*: mesoerythritol, erythritol, and 1,4-dideoxy-1,4-imino-D-arabinitol;* quinone*: 4-bromo-3,5-cyclohexadiene-1,2-dione;* heterocycles*: naltrindole, scopoletin, and piperonyl butoxide;* aromatic*: benzidine;* cyclic*: beta-2,3,4,5,6-pentachlorocyclohexanol;* polycyclic*: corrinoid;* fatty acids*: 4-hydroxy-valeric acid, 8S-hydroxy-hexadecanoic acid, 2,3-dinor-TXB2, arachidic acid (d3), and 7-hydroxy tetranor iloprost;* fatty acyls*: n-valeryl acetic acid, 12-hydroxy-10-octadecynoic acid, and 9,10-dioxo-octadecanoic acid;* fatty alcohol*: 2,2,9,9-tetramethyl-decan-1,10-diol and dolichyl phosphate;* sterol lipids*: cholestan-3*α*-ol,3*α*,6*α*,7*α*,12*α*-tetrahydroxy-5*β*-cholan-24-oic acid, alpha-tocopheronic acid, and (24R)-1*α*,24-dihydroxy-22-oxacholecalciferol;* glycerides*: 1-monopalmitin;* terpenes and others*: tetrahydrocortisone, cucurbitacin A, and C25:3 6,7-epoxy highly branched isoprenoid;* amine*: 1-naphthylacetylspermine;* amino acid*: Tyr Met, Thr Ser Asn, Arg Ser, N-acetyl-leucyl-leucine, Ala His Ala, His Arg Asn, Thr Gln Tyr, Thr Tyr Tyr, Gln His Gly, and Leu Ala Arg;* amide*: fenothiocarb sulfoxide.

On the other hand, adduct in LC-MS in negative mode provided the following:* nucleoside*: azacitidine;* glucuronic acid*: isovalerylglucuronide;* carbohydrate acid:* arabinonic acid;* polysaccharides*: levan;* fatty acid*: 2-hydroxy-6-oxo-6-(2-hydroxyphenyl)-hexa-2,4-dienoate;* steroids*: fluocinolone and dexamethasone phosphate;* fatty acyls*: 18-hydroxy-9S,10R-epoxy-stearic acid, 7-keto-stearic acid, 2,4-dimethyl-tetradecanoic acid, and DL-9-hydroxy stearic acid;* terpenes:* antirrhinoside and monotropein;* heterocyclic compounds*: etanidazole, 5-acetylamino-6-amino-3-methyluracil, nifuradene, 3,7-dimethyluric acid, xanthine, hypoxanthine, scopoletin, rutin, and dioclein;* alkaloid*: atalanine;* amino acid derivatives*: fructoselysine 6-phosphate.

### 3.2. Quantification of Scopoletin and Rutin in MMC by LCMS/MS Analysis

The concentration of scopoletin and rutin in MMC was found to be 18.95 *μ*g/mg and 1.66 *μ*g/mg, respectively.

### 3.3. Effect of MMC, Scopoletin, and Rutin Hydrate on DA- or NA-Evoked Contractile Response

**Figure 2:**
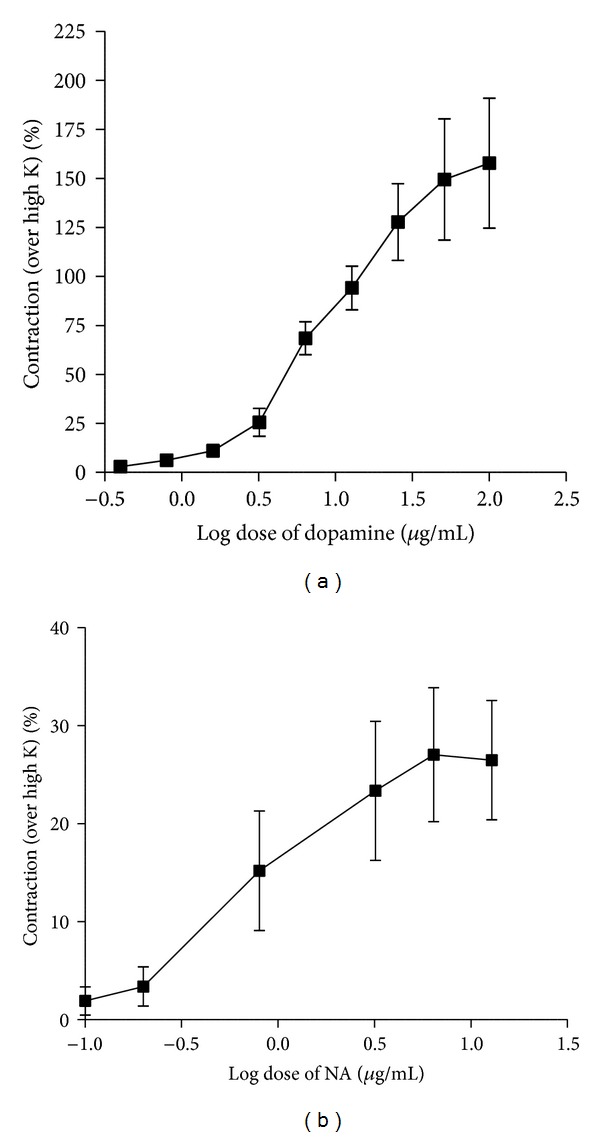
Log dose-response curve of (a) dopamine and (b) noradrenaline (NA) employing isolated rat vas deferens preparation (*n* = 5-6).

Dopamine and noradrenaline produced concentration-dependent contractions of epididymal segments of the isolated rat vas deferens preparation are shown in Figures 2(a) and 2(b).

**Figure 3:**
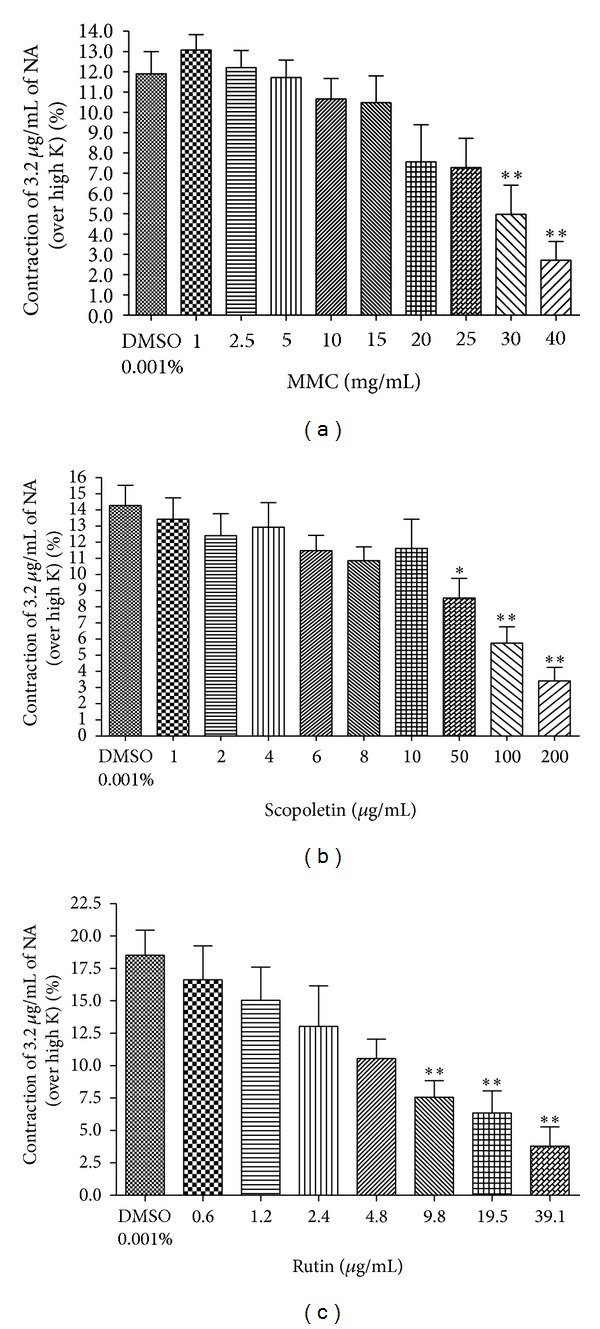
Effect of (a) MMC (1–40 mg/mL), (b) scopoletin (1–200 *μ*g/mL), and (c) rutin hydrate (0.6–39.1 *μ*g/mL) on NA- (3.2 *μ*g/mL) evoked contraction expressed as % contraction over 80 mM High K. *P < 0.05 and **P < 0.01 compared to DMSO (0.001% v/v); *n* = 5.

**Figure 4:**
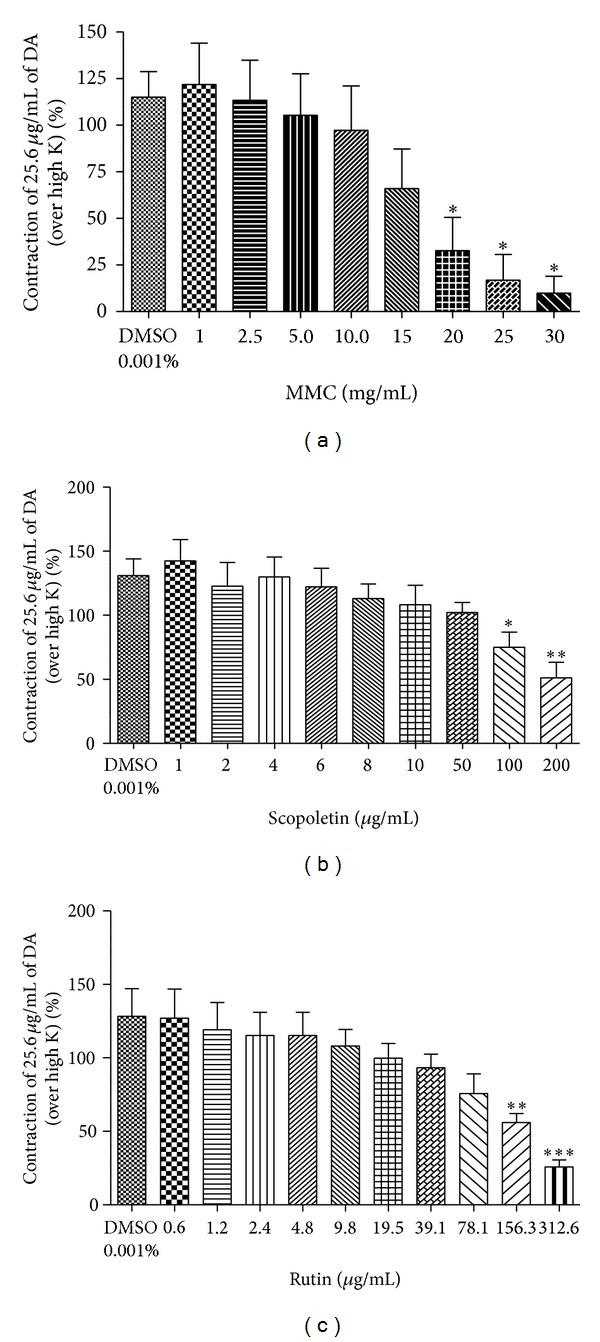
Effect of (a) MMC (1–30 mg/mL), (b) scopoletin (1–200 *μ*g/mL), and (c) rutin hydrate (0.6–312.6 *μ*g/mL) on dopamine- (25.6 *μ*g/mL) evoked contraction in isolated rat vas deferens preparations expressed as % contraction over 80 mM High K. *P < 0.05, **P < 0.01, and ***P < 0.001 compared to DMSO (0.001% v/v); *n* = 5.

After incubation of the tissue preparation with MMC (1–40 mg/mL), scopoletin (1–200 *μ*g/mL), and rutin hydrate (0.6–312.6 *μ*g/mL) for 5 min, the contractile responses to the selected submaximal dose of NA (3.2 *μ*g/mL) and DA (25.6 *μ*g/mL) were significantly reduced in dose-dependent manner (Figures 3(a), 3(b), and 3(c) and Figures 4(a), 4(b), and 4(c)).

### 3.4. Log Dose-Response Curve of NA/DA in Absence and in Presence of MMC

**Figure 5:**
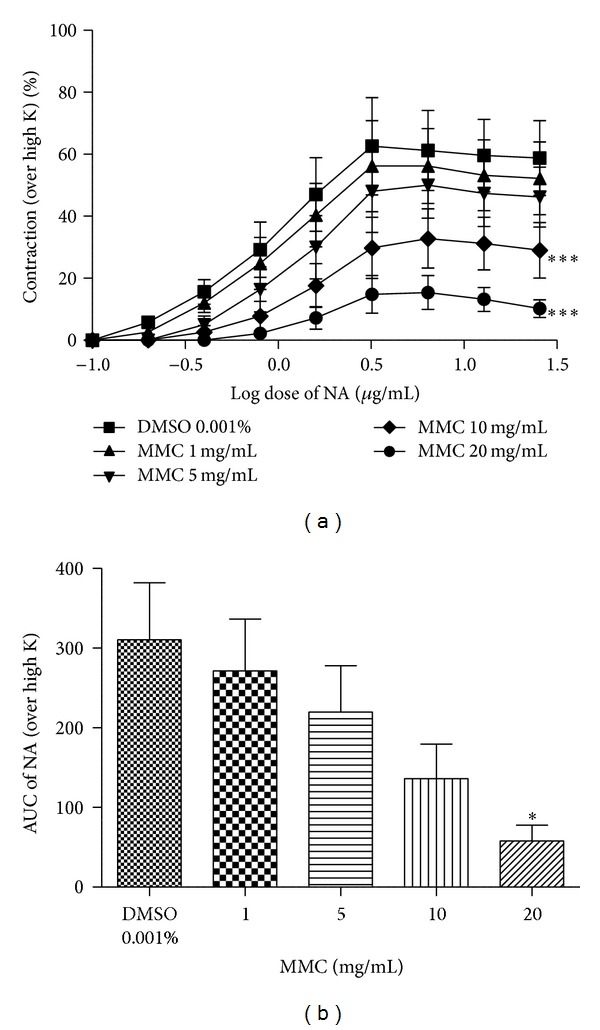
Log dose-response curve of (a) noradrenaline (NA) and (b) area under curve in absence and in presence of MMC, expressed as % contraction over 80 mM High K. *P < 0.05 and ***P < 0.001 compared to DMSO (0.001% v/v); *n* = 5.

**Figure 6:**
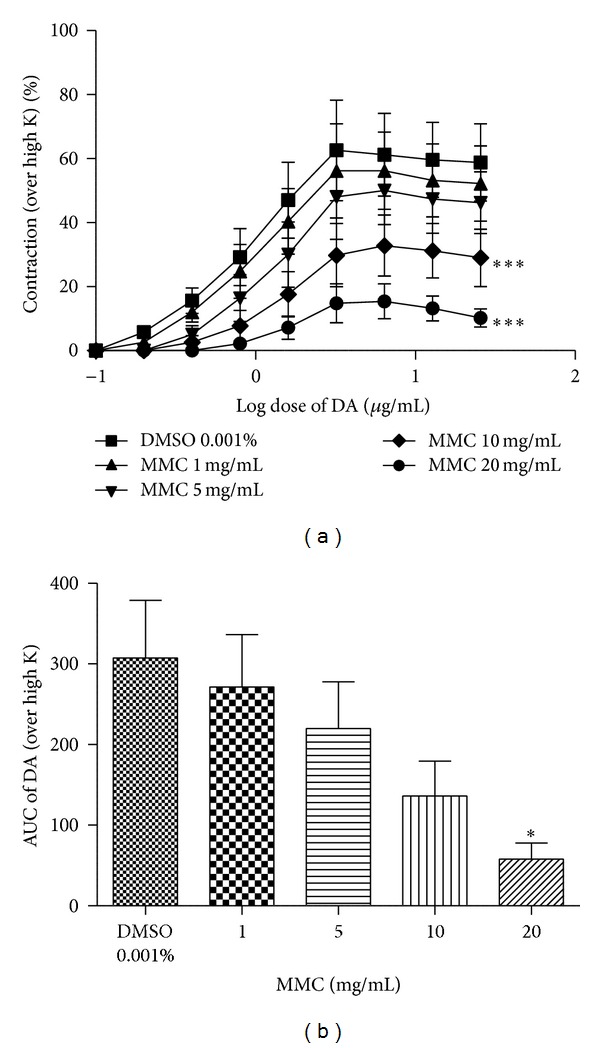
Log dose-response curve of (a) dopamine (DA) and (b) area under curve in absence and in presence of MMC, expressed as % contraction over 80 mM High K. *P < 0.05 and ***P < 0.001 compared to DMSO (0.001% v/v); *n* = 5.

As shown in Figures 5(a), 5(b), 6(a), and 6(b), the log dose-response curves of NA and DA were dose-dependently shifted rightward in the presence of MMC (1–20 mg/mL). The maxima of the agonists (NA/DA) curves were also progressively depressed by MMC in a dose-dependent manner. The values of the calculated area under the curve (AUC) of NA and DA were significantly decreased by MMC dose-dependently.

### 3.5. Effect of MMC on 80 mM High K-Evoked Contractile Response

MMC (1–40 mg/mL) did not alter 80 mM High K-evoked contractile response in rat vas deferens preparations (data not shown).

### 3.6. Effect of Haloperidol or Prazosin HCl on DA or NA-Evoked Contractile Response

**Figure 7:**
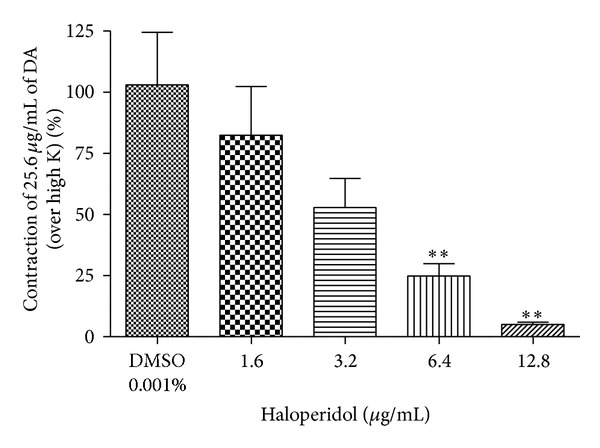
Effect of haloperidol (1.6–12.8 *μ*g/mL) on dopamine- (25.6 *μ*g/mL) evoked contraction on isolated rat vas deferens preparation. **P < 0.01 compared to DMSO (0.001% v/v); *n* = 4.

While incubating the vas deferens preparation with haloperidol (1.6–12.8 *μ*g/mL) for 5 min, the contractile responses to the selected submaximal dose of DA (25.6 *μ*g/mL) were significantly reduced in dose-dependent manner (Figure 7). Prazosin HCl (0.01 *μ*g/mL) showed 100% inhibition on NA response (data not shown).

### 3.7. Effect of High Doses of MMC, Scopoletin, and Rutin* Per Se* on Contractility of Rat Vas Deferens

**Figure 8:**
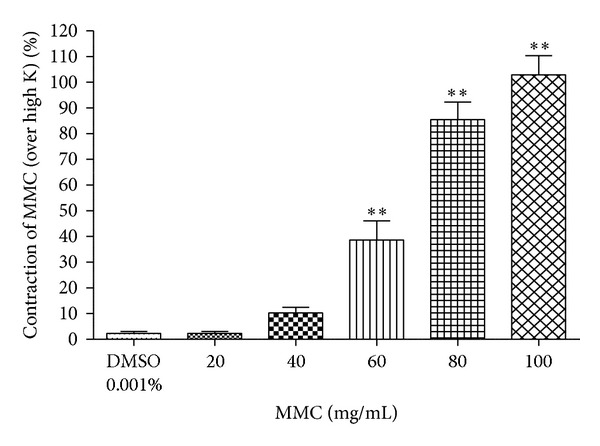
Contraction evoked by MMC (20–100 mg/mL)* per se* expressed as % contraction over 80 mM High K. **P < 0.01 compared to DMSO (0.001% v/v); *n* = 4.

Remarkably, MMC* per se* at higher doses (60, 80, and 100 mg/mL) significantly increased the contractility of vas deferens in a dose-dependent manner (Figure 8). However, the bioactive principles of MMC, scopoletin, and rutin, respectively, at higher doses (0.5–5 mg/mL) did not show any contractile response (data not shown) in rat vas deferens.

### 3.8. Effect of Haloperidol or Prazosin HCl on High Dose MMC-Evoked Contractile Response

**Figure 9:**
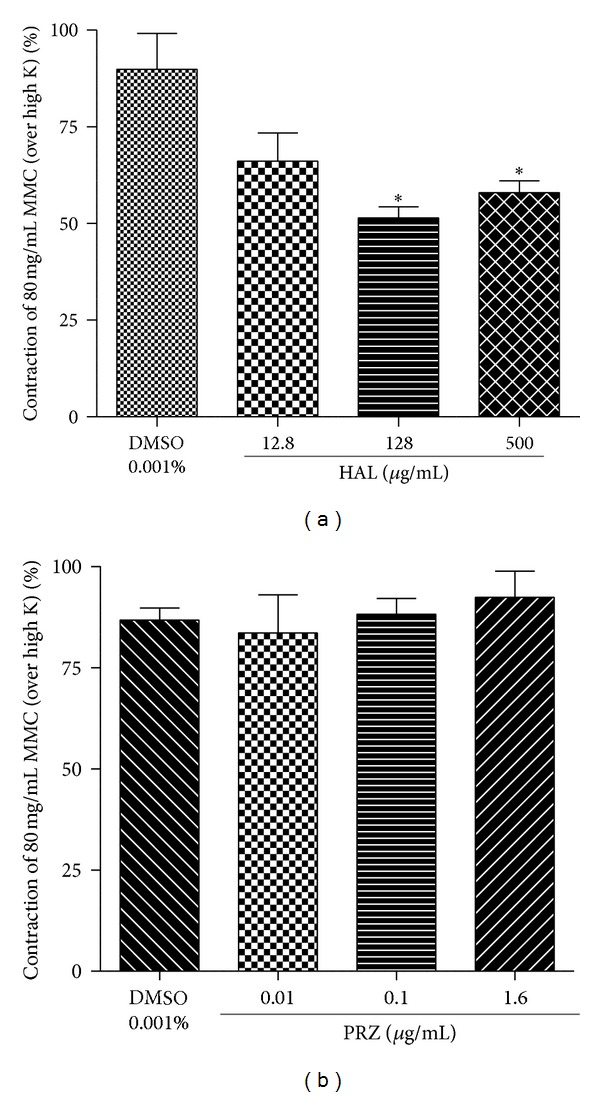
Effect of haloperidol (a) (HAL 12.8, 128, and 500 *μ*g/mL) and (b) prazosin (PRZ 0.01, 0.1, and 1.6 *μ*g/mL) on MMC (80 mg/mL)* per se* induced contractility expressed as % contraction over 80 mM High K. *P < 0.05 compared to DMSO (0.001% v/v); *n* = 4.

Haloperidol (12.8–500 *μ*g/mL) significantly decreased the contractility induced by high dose MMC (80 mg/mL) (Figure 9(a)). On the other hand, prazosin HCl even at higher concentration (1.6 *μ*g/mL) pretreatment could not alter the contractile response of high doseMMC (Figure 9(b)).

## 4. Discussion

The isolated rodent vas deferens is an established research model for functional and molecular investigation of normal and altered autonomic (sympathetic) neuroeffector coupling [24]. It has also been suggested that dopamine may be a neurotransmitter in the rat vas deferens and that excitatory dopamine receptors are located on the smooth muscle [25–27]. Boadle-Biber and Roth (1975) reported that a high proportion of newly formed catecholamine in the rat vas deferens was present as dopamine as compared to noradrenaline [28]. Relja et al. (1982) reported that dopamine receptors are present in rat vas deferens, based on the binding of [^3^H] haloperidol to the plasma membranes [29]. Besides that, Ohmura et al. (1992) indicated the occurrence of two distinct *α*_1_-adrenoceptor subtypes in the rat vas deferens [30].

The preliminary phytochemical analysis of the methanol extract of* M. citrifolia* showed the presence of carbohydrate, alkaloids, saponin, glycosides, tannins, flavonoids, and steroids [31]. Phytochemical studies using high performance liquid chromatographic (HPLC) fingerprint profile of the MeOH extracts of noni fruit revealed three major peaks representing scopoletin, rutin, and quercetin together with several minor peaks. These are major bioactive constituents of noni responsible for various pharmacological activities [32, 33]. The present phytochemical analysis results are consistent with earlier findings and confirmed the presence of scopoletin and rutin (retention times: 14.51 and 15.86 min, resp.). Furthermore, we estimated the concentration of these constituents in MMC using LCMS/MS analysis and it has been found that MMC is enriched with scopoletin (18.95 *μ*g/mg) and rutin (1.66 *μ*g/mg). Hence, in the present study, scopoletin and rutin were used to establish its interaction with dopaminergic and noradrenergic systems.

In the present study, we observed that MMC (<40 mg/mL), scopoletin (100, 200 *μ*g/mL), and rutin hydrate (156.3, 312.6 *μ*g/mL) caused a significant concentration-dependent inhibition of contraction evoked by dopamine in the isolated rat vas deferens preparations, thus indicating antidopaminergic activity of MMC, scopoletin, and rutin hydrate (Figures 4(a), 4(b), and 4(c)). It has been observed that MMC [1–20 mg/mL; equivalent to scopoletin (18.95–379 *μ*g/mL) and rutin (1.66–33.2 *μ*g/mL)] dose-dependently decrease the slope as well as maxima of the agonists (DA/NA) curves (Figures 5(a), 5(b), 6(a), and 6(b)) and suggested that the antagonistic effect of MMC (<40 mg/mL) on dopaminergic and noradrenergic system might be irreversible. Furthermore, MMC (<40 mg/mL) did not alter the contraction induced by high K (80 mM KCl) in rat vas deferens. This observation excludes the possibility of the observed inhibitory effect of MMC (<40 mg/mL) on rat vas deferens being due to direct muscle relaxing effect of MMC. The reference drug, haloperidol (dopamine D_2_ receptor blocker, 1.6–12.8 *μ*g/mL), also showed dose-dependent inhibition on dopamine-induced contractile response in rat vas deferens. Conversely, MMC* per se* at higher doses (60, 80, and 100 mg/mL) exhibited significant dose-dependent contractile response. Interestingly, contractility evoked by MMC* per se* was significantly inhibited by high doses of haloperidol (128–500 *μ*g/mL). Thus, the present results have demonstrated the biphasic effect of MMC on dopaminergic system, with dopaminergic antagonistic effect at lower concentrations (<40 mg/mL) and dopaminergic agonistic effect at higher concentrations (>60 mg/mL). However, the main bioactive principles of noni, scopoletin, and rutin* per se* at higher doses (0.5–5 mg/mL) did not show any contractile response in rat vas deferens. Therefore, it can be postulated that the antidopaminergic effects of MMC might be mediated by its active principles, scopoletin and rutin. Nevertheless, these active principles might not be involved in the high dose contractile response of MMC* per se*. There is a possibility that some other bioactive phytoconstituents of MMC could be involved in the high dose contractile response of MMC.

*M. citrifolia* has been used as an antiemetic agent in patients considered with high risk for postoperative nausea and vomiting (PONV) after various types of surgery. The 600 mg dose of noni extract (equivalent to 20 g of dried noni fruit/8.712 *μ*g of scopoletin) was the minimum dose that could effectively reduce the incidence of postoperative nausea in the early postoperative period [34]. However, these studies could not reveal the possible mechanism of action of* M. citrifolia* for its antiemetic action. In our recent published report, we suggested the antipsychotic and antiemetic activities of* M. citrifolia* might be mediated by antidopaminergic mechanism [6]. Another recent report on neurochemical analysis revealed the alterations in the monoaminergic system in the noni-treated rats (1 mL/day, p.o., for 15 days), compared to the control rats. It was found that the reduction in these neurotransmitters including noradrenaline in the amygdala and the hippocampus, serotonin in the amygdala, DOPAC in the hippocampus and the substantia nigra, and HVA in the substantia nigra of the noni group might be responsible for anxiolytic effects of noni juice [8]. Conversely, it has been demonstrated that ethyl acetate fraction of crude methanol extract of* M. citrifolia* at a daily dose of 200 and 400 mg/kg which was administered to the rats for 15 days significantly increased the brain levels of serotonin, dopamine, and noradrenaline [7]. This opposing effect of noni on monoamine levels in two different studies could be due to the difference in the doses of noni used for these studies. Lower doses of noni might inhibit monoaminergic system whereas higher concentrations could stimulate monoaminergic system. The enhancement in brain monoamines after 15-day treatment of ethyl acetate fraction of crude methanol extract of* M. citrifolia* might be due to the presence of enrichment of active constituents in the ethyl acetate fraction. The higher concentration of active constituents in the ethyl acetate fraction when compared to crude MMC could activate the monoaminergic system. The present results are in good agreement with these earlier findings; that is, MMC can act as a dopaminergic antagonist at lower concentrations (<40 mg/mL) and as a dopaminergic agonist at higher dose (>60 mg/mL). However further receptor-ligand binding assays are necessary to confirm the biphasic effects of* M. citrifolia* on dopaminergic system.

In the present study, MMC showed antiadrenergic effect at lower concentrations [<30 mg/mL; equivalent to scopoletin (<568.5 *μ*g/mL) and rutin (<49.8 *μ*g/mL)] in the isolated rat vas deferens preparation. Besides that, scopoletin (50, 100, 200 *μ*g/mL) and rutin hydrate (9.8, 19.5, 39.1 *μ*g/mL) caused a significant concentration-dependent inhibition of contraction evoked by noradrenaline (Figures 3(a), 3(b), and 3(c)). The reference drug, prazosin HCl (adrenergic *α*_1_ receptor blocker), completely reversed the noradrenaline-evoked contractility in the isolated rat vas deferens even at lower concentration of 0.01 *μ*g/mL. A recent research report suggested that the spasmolytic and vasodilator effects of* M. citrifolia* root extract are mediated possibly through blockade of voltage-dependent calcium channels and release of intracellular calcium and justified traditional medicinal usage of* M. citrifolia* in diarrhoea and hypertension [11]. These studies also demonstrated the vasorelaxant activity of* M. citrifolia* by inhibiting contractile response of *α*_1_ adrenoceptors agonist, phenylephrine (1 *μ*M), in endothelium-intact rat aortic preparations [11]. The present results are consistent with these earlier findings and suggest that the antihypertensive effect of* M. citrifolia* might be partially mediated by *α*_1_ adrenoceptors blocking effect. The present study results also revealed the antiadrenergic activity of scopoletin and rutin, respectively, in rat vas deferens. Therefore, it can be postulated that *α*_1_ adrenoceptors blocking effect of MMC could be mediated by its bioactive principles, scopoletin and rutin. An earlier report revealed the predominance of *α*-adrenoceptors in the testicular (epididymal) segment of the vas deferens compared with the urethral (prostatic) segment [35]. A nerve-stimulation evoked postjunctional receptor for NA is the *α*_1A_-adrenoceptor, acting via inositol trisphosphate (IP_3_) leading to increase in intracellular Ca^2+^ and the slow component of nerve-mediated contraction [36]. However, the possibility that there are differences between neurogenic and exogenously applied NA has been raised [37]. They showed that contractions to exogenous NA involved both *α*_1A_ and *α*_2A/D_ adrenoceptors. Hence the antiadrenergic effect of MMC (<30 mg/mL) and its bioactive principles, scopoletin and rutin, could be mediated by both *α*_1A_ and *α*_2A/D_ adrenoceptors. The contractile response of high dose of MMC (80 mg/mL) was not inhibited by *α*_1_-adrenoceptor blocker, prazosin, even at higher concentration (1.6 *μ*g/mL). Therefore, the contractility response of high dose of MMC might not be mediated through adrenergic mechanism. The biphasic effect of MMC observed in the present study using rat vas deferens preparation could be exhibited only at the dopaminergic system.

## 5. Conclusion

In conclusion, MMC showed biphasic effect on dopaminergic system, that is, antidopaminergic effect at lower dose (<40 mg/mL) and dopaminergic agonistic effect at higher dose (>60 mg/mL) in the isolated rat vas deferens preparation. Additionally, MMC (<30 mg/mL) showed the antiadrenergic activity in the rat vas deferens. Furthermore, antidopaminergic and antiadrenergic activities of scopoletin (<200 *μ*g/mL) and rutin hydrate (<312.6 *μ*g/mL), respectively, have been established. It has been postulated that the bioactive principles of noni, scopoletin and rutin, could be responsible for the antidopaminergic and antiadrenergic activities of MMC. However, the mechanism of high dose contractile response of MMC on rat vas deferens could not be explained in the present study. Further,* in vivo* animal behaviour studies are warranted to confirm the biphasic effect of noni on dopaminergic system. Studies in this direction are currently under way in our laboratory.
